# Microminutinin, a Fused Bis-Furan Coumarin from *Murraya euchrestifolia*, Exhibits Strong Broad-Spectrum Antifungal Activity by Disrupting Cell Membranes and Walls

**DOI:** 10.3390/plants14213392

**Published:** 2025-11-05

**Authors:** Duan-Tao Cao, Ying-Juan Yao, Xiao-Xiang Fu, Wen-Wu Song, Xin-Yuan Liu, Peng Zhang, Qing-Hong Zhou, Bao-Tong Li, Wen-Wen Peng

**Affiliations:** 1 The Laboratory for Phytochemistry and Botanical Pesticides, College of Agriculture, Jiangxi Agricultural University, Nanchang 330045, China; cdtagri@jxau.edu.cn (D.-T.C.);; 2 Jiangxi Province Key Laboratory of Vegetable Cultivation and Utilization, Jiangxi Agricultural University, Nanchang 330045, China; 3 Institute of Agricultural Applied Microbiology, Jiangxi Academy of Agricultural Sciences, Nanchang 330200, China

**Keywords:** botanical fungicides, plant secondary metabolites, tea grey blight, bioassay-guided fractionation, plant pathogenic fungi

## Abstract

Plant fungal diseases pose a serious threat to crop production and safety, and natural products are one of the important directions for the development of new green fungicides. This study found that the extract of *Murraya euchristifolia* had significant antifungal activity, and a main antifungal coumarin (1) was isolated by bioassay-guided fractionation. The structure of 1 was identified by NMR and MS spectroscopic data as a fused bis-furan coumarin (microminutinin) which was first isolated from the *Murraya* genus and exhibited strong broad-spectrum antifungal activity against eight plant pathogenic fungi from different families and genera. The EC_50_ value of 1 (11.33 μg/mL) against *Pestalotiopsis theae* (the most sensitive to 1) was slightly higher than that (7.03 μg/mL) of the positive drug (80% carbendazim WP), indicating that 1 has the potential to serve as a lead compound for botanical fungicides. The bioassay results against *P. theae* in vivo indicated that 1 also has the potential for field application. Scanning electron microscopy and optical microscopy revealed that 1 disrupted the morphological structure of mycelium, causing hyphae to twist, shrink, and even crack and severely reducing hyphal branching. Furthermore, propidium iodide staining proved that microminutinin destroyed the integrity of the cell membrane, causing leakage of cellular components. In addition, calcofluor white staining and chitin content changes illustrated that microminutinin disrupted the cell wall structure. This research provides compound sources and a theoretical basis for the development of botanical fungicides.

## 1. Introduction

Plant secondary metabolites (PSMs) have complex and diverse chemical structures and extensive biological activities. They are essential compound resources for discovering lead compounds and developing new pesticides [1,2,3]. At present, many commercial pesticides come directly or indirectly from PSMs, such as pyrethroids and neonicotinoid insecticides [4,5]. Therefore, the discovery of highly active compounds from PSMs has always been a focus of new pesticide development.

Among the numerous PSMs, coumarins are one of the major categories. They not only show assorted medical biological activities, for example, antibacterial, antitumor, antioxidant, anti-inflammatory, anticoagulant, and other medicinal biological activities, but also act as one of the crucial phytoalexins for plant pathogens and a response to biological and abiotic stressors [6,7,8]. Some studies have shown that coumarins can be biosynthesized from α-pyrone and benzene rings through the phenylpropane pathway in many plants and accumulate as a response to bacterial, fungal, viral, and oomycephalic infections, playing a role in plant antitoxins [9,10].

In nature, coumarins are mainly distributed in Umbelliferae, Leguminosae, Compositae, Solanaceae, Daphne, Rutaceae, and other plants and are also found in animal and microbial metabolites [11]. They are often classified into simple coumarins, furan coumarins, pyranocoumarins and other coumarins based on the substituents and positions on the parent nucleus [12,13]. Heretofore, more than 1000 coumarins and their derivatives have been found in more than 800 plants or microorganisms [11].

Recent studies have found that coumarins have excellent antibacterial and antifungal activities [14,15]. The antibacterial effect of daphnetin is mainly achieved by inhibiting the gene expression of *xpsR*, *epsE*, *epsB*, and *epsM* to suppress the production of extracellular polysaccharides and biofilm formation in *Ralstonia solanacearum* [14]. The induction effect of scopoletin significantly increases the resistance of young tobacco leaves to *Alternaria alternata* compared to mature leaves [15]. Scopolin, coniferin and syringin can be rapidly produced and accumulated in *Arabidopsis* roots after being infected by the oomycetes pathogen *Pythium sylvaticum* and produce lignin that reinforces cell walls and the antimicrobial component scopoletin [16].

*Murraya euchrestifolia* Hayata is a plant in the Rutaceae family, belonging to the *Murraya* genus. It is distributed in TaiWan, GuangDong, HaiNan, GuangXi Province and other places in China [17]. Its branches and leaves are used as medicine in folk medicine, with the effects of dispelling wind, promoting blood circulation, and reducing inflammation and pain [18]. Previous antibacterial experiments have shown that the essential oil of *M. euchrestifolia* has a strong broad-spectrum antibacterial effect [17,18]. Preliminary phytochemistry investigations have elucidated that the main components of *M. euchrestifolia* are carbazole alkaloids, coumarins, phenylpropanoids, and flavonoids [17,19,20,21]. However, to our knowledge, there are currently no reports on the agricultural antifungal activities of *M. euchrestifolia*, and it is also unknown whether coumarins play a major role in the antifungal activity of extracts from *M. euchrestifolia*. Therefore, our aim is to combine bioactivity guided solvent extraction, column chromatography (CC), and high-performance liquid chromatography (HPLC) to reveal the antifungal active coumarins in the extract of *M. euchrestifolia*.

In this study, based on the preliminary experimental results, the methanol extract of *M. euchrestifolia* showed strong inhibitory effects on 12 fungal pathogens, with an especially prominent inhibitory effect on *Pestalotiopsis theae* and *Botryosphaeria dothidea*. Therefore, *P. theae* and *B. dothidea* were used as test fungi for bioactivity-guided separation. Using a 254 nanometer UV lamp, 6% H_2_SO_4_ ethanol solution as a color reagent, and HPLC to guide the isolation of coumarins, the structures of the isolates were identified through mass spectroscopy (MS) and nuclear magnetic resonance (NMR) analyses. In addition, the broad-spectrum antifungal activities of the most effective coumarin were assessed against eight common plant pathogenic fungi from four families and six genera. Furthermore, the inhibitory effect of active coumarin on the pathogenicity of tea grey blight (TGB) caused by *P. theae* was evaluated on fresh tea leaves. Finally, the mechanism by which active coumarin inhibits fungi was analyzed and elucidated using physiological and biochemical experimental results.

## 2. Results

### 2.1. Antifungal Coumarin Guided Isolation

#### 2.1.1. The Antifungal Activity of *M. euchrestifolia* Methanol Extract (ME)

**Figure 1:**
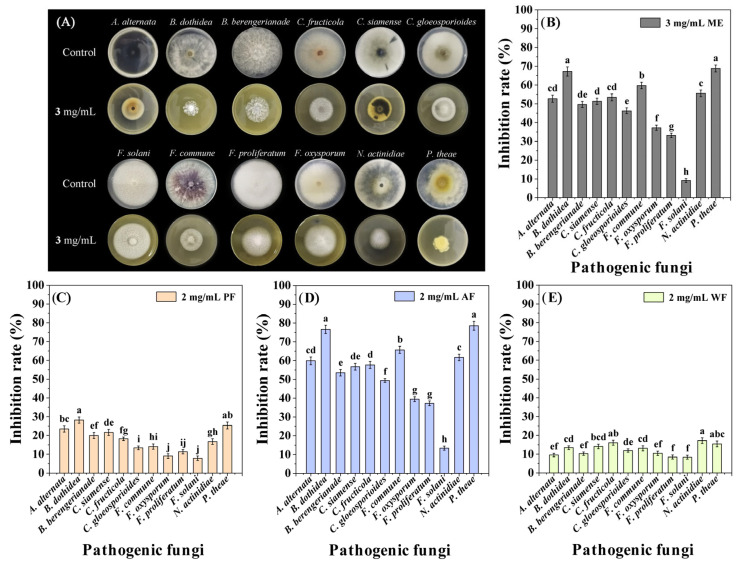
Inhibitory effect of ME, PF, AF, and WF on the mycelial growth of 12 pathogenic fungi. (**A**,**B**): The antifungal activity of ME; (**C**–**E**): The antifungal activities of PF, AF, and WF. Data are the mean of three replicates, and the different letters on the columns indicate significant differences among pathogenic fungi by analysis of variance with Tukey’s test at *p* = 0.05.

The antifungal activity of ME was evaluated through the mycelial growth rate method [22]. The test results showed that ME exhibited extensive inhibitory effects on 11 tested fungi except *Fusarium solani* at 3 mg/mL, with inhibition rates (IRs) ranging from 33.15 to 68.80% (Figure 1A,B) [Supporting Information (SI) Table S1], indicating that ME had broad-spectrum antifungal activity.

#### 2.1.2. Antifungal Fraction Screening of ME

The aqueous suspension of ME was subjected to liquid-liquid extraction using petroleum ether (PE) and ethyl acetate (EA) and was roughly divided into three fractions [PE extraction fraction (PF), EA extraction fraction (AF), and aqueous phase (WF)]. The screening results of the antifungal fraction indicated that AF had the strongest antifungal activity among the three fractions, in the order of AF > PF > WF (Figure 1C,E). In addition, among the 12 pathogenic fungi, *P. theae* and *B. dothidea* were most sensitive to AF, with IRs of 78.51 and 76.62% at a concentration of 2 mg/mL, respectively (Figure 1D) (SI Table S1). So, in the subsequent isolation process, *P. theae* and *B. dothidea* were used as activity indicator fungi.

#### 2.1.3. Isolation of Antifungal Coumarin

**Figure 2:**
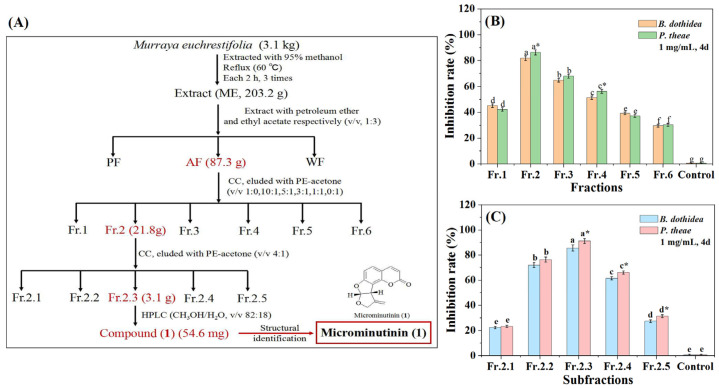
Procedure for antifungal coumarin guided isolation from the *M euchrestifolia* extract. (**A**): Separation process diagram of 1; (**B**): Antifungal activity of Fr.1–Fr.6 against *P. theae* and *B. dothidea*; (**C**): Antifungal activity of Fr.2.1–Fr.2.5 against *P. theae* and *B. dothidea*. The red part in (**A**) represent fractions that had high antifungal activity. Data are the mean of three replicates, the different letters on the columns represent significant differences among fractions (or subfractions) by analysis of variance with Tukey’s test at *p* = 0.05. “*” on the columns represent significant differences between pathogenic fungi by analysis of variance with an independent sample *t*-test (*p* < 0.05).

The isolation process was guided by antifungal assays combined with thin-layer chromatography (TLC) and HPLC (at 254 nm) (Figure 2A). AF was further subjected to activity-guided isolation on a silica gel column to obtain coumarin with the highest antifungal activity and eluted with a mixture of PE and acetone to give six fractions (Fr.1–Fr.6). We subsequently compared their antifungal activities against *P. theae* and *B. dothidea*, as well as their coumarin chromatographic characteristics (strong blue fluorescence under 254 nm ultraviolet lamp). The results showed that Fr.2 had the most significant inhibitory effect (*p* < 0.05) on two pathogenic fungi at 1 mg/mL (with IRs of 86.28 and 81.92%, respectively) (Figure 2B) (SI Table S2), and had strong blue fluorescence under 254 nm ultraviolet lamp. Therefore, Fr.2 was further subjected to guided isolation on a silica gel column to yield five subfractions (Fr.2.1–Fr.2.5). Further based on antifungal assays and coumarin detection, Fr.2.3 had the strongest antifungal activity against *P. theae* and *B. dothidea* at 1 mg/mL (with IRs of 91.13 and 85.63%, respectively) (Figure 2C) (SI Tables S4 and S3), and exhibited strong blue fluorescence under 254 nm ultraviolet lamp. Therefore, the main antifungal coumarin (1) of Fr.2.3 was purified using HPLC.

### 2.2. Structural Identification of 1

Through comprehensive analysis of the ^1^H NMR spectra, ^13^C NMR spectra, and electrospray ionization mass spectrometry (ESI-MS) data of 1, it has been identified as microminutinin (Figure 2A), a fused bis-furan coumarin. The details are as follows: crystals from MeOH, mp 114–116; ESI-MS, *m*/*z*: 243 [M + H]^+^; High resolution electrospray ionization mass spectroscopy (HR-ESI-MS) *m*/*z*: 243.0656 (Calculated for: [C_14_H_10_O_4_ + H]^+^, 243.0657). ^1^H-NMR (400 MHz, CDCl_3_): *δ*_H_: 7.65 (^1^H, d, *J* = 9.6 Hz, H-4), 7.32 (^1^H, d, *J* = 8.3 Hz, H-5), 6.78 (^1^H, d, *J* = 8.3 Hz, H-6), 6.55 (^1^H, d, *J* = 5.8 Hz, H-2′), 6.23 (^1^H, d, *J* = 9.6 Hz, H-3), 5.73 (^1^H, br s, H-6′a), 5.19 (^1^H, br s, H-6′b), 4.66 (^1^H, dd, *J* = 5.8, 1.6 Hz, H-3′), 4.49 (^1^H, d, *J* = 12.4, 1.6 Hz, H-5′a), 4.41 (^1^H, ddd, *J* = 12.4, 1.6, 1.0 Hz, H-5′b); ^13^C-NMR (100 MHz, CDCl_3_): *δ*_C_: 162.2 (C, C-7), 160.5 (C, C-2), 151.5 (C, C-9), 144.3 (C, C-4′), 140.0 (CH, C-4), 129.5 (CH, C-5), 113.7 (CH, C-2′), 113.6 (C, C-10), 113.4 (C, C-8), 112.6 (CH, C-3), 109.6 (CH_2_, C-6′), 106.9 (CH, C-6), 70.8 (CH_2_, C-5′), 48.6 (CH, C-3′). The above data were in agreement with the data reported by Rahmani et al. [23]; Therefore, 1 was identified as microminutinin. The ^1^H NMR, and ^13^C NMR spectra are shown in the Supporting Information (SI Figures S1–S4).

### 2.3. The Antifungal Activity of Microminutinin In Vitro

**Figure 3:**
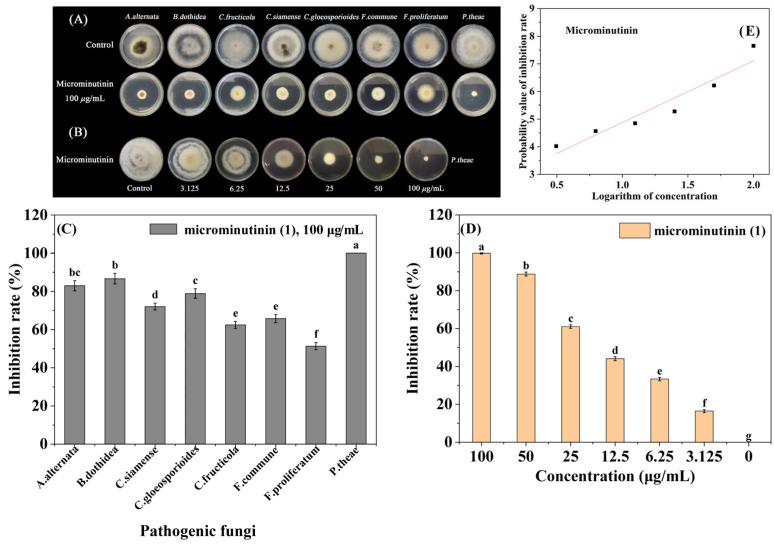
The broad-spectrum antifungal activity of microminutinin in vitro. (**A**,**C**): Antifungal activity of microminutinin against 8 plant pathogenic fungi; (**B**,**D**): Antimicronin’s antifungal activity against *P. theae*; (**E**): The toxicity regression curve of microminutinin on *P. theae*. Data are the mean of three replicates, and the different letters on the columns indicate significant differences among pathogenic fungi (in (**C**)) or treatment (in (**D**)) by analysis of variance with Tukey’s test at *p* = 0.05.

Based on the screening results of antifungal activity in guided separation, 8 sensitive pathogenic fungi (*Alternaria alternate*, *B. dothidea*, *Colletotrichum fructicola*, *Colletotrichum siamense*, *Colletotrichum gloeosporioides*, *Fusarium commune*, *Fusarium proliferatum*, *P. theae*) were selected for broad-spectrum antifungal activity assays of microminutinin. The results showed that microminutinin had significant inhibitory activity (*p* < 0.05) against 8 pathogenic fungi at 100 μg/mL, with IRs ranging from 51.32 to 100.00% (Figure 3A–C) (SI Table S4), indicating that microminutinin had broad-spectrum antifungal activity. Due to *P. theae* being the most sensitive to microminutinin with a 100% IR at 100 μg/mL, the EC_50_ values (half-maximal effective concentration) and antifungal mechanism of microminutinin was further studied on *P. theae*.

According to the toxicity regression equations (Table 1) of microminutinin and 80% carbendazim WP, the EC_50_ values of microminutinin and 80% carbendazim WP against *P. theae* were calculated to be 11.33 and 7.03 μg/mL, respectively. The results indicated that although the inhibitory effect of microminutinin on *P. theae* was slightly weaker than that of 80% carbendazim WP, it still has the potential to be used as a lead compound to develop botanical fungicides.

### 2.4. The Suppressive Effect of Microminutinin on the TGB Pathogenicity In Vivo

**Figure 4:**
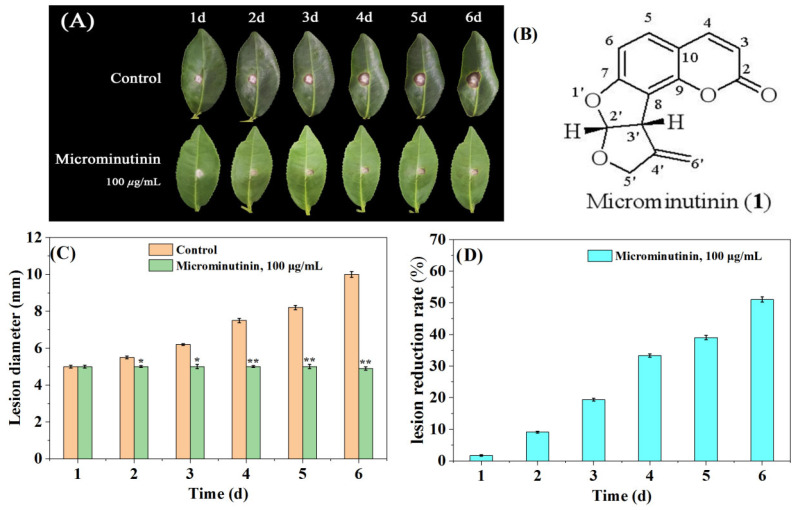
The suppressive effect of microminutinin on the pathogenicity of TGB. (**A**): The inhibitory effect of microminutinin on TGB; (**B**): The chemical structure of microminutinin; (**C**): TGB lesion diameter after treatment with microcantinin; (**D**): Reduction rate of TGB lesions after treatment with microcantinin. Data are the mean of three replicates, “*” on the columns represent significant difference from control (*p* < 0.05) by analysis of variance with an independent sample *t*-test, and “**” represent significant difference from control (*p* < 0.01).

In general, the actual dose used to control fungal diseases in crops is much higher than in vitro experiments. Therefore, this study used a concentration of approximately 10 × EC_50_ to evaluate the inhibitory effect of microminutinin on TGB. Briefly, fresh tea leaves (“Luyun-1”) were used as targets for *P. theae* infection. From the second day after treatment, compared with the control, microminutinin showed significant suppressive effects (*p* < 0.05) on the colonial expansion of TGB at concentrations of 100 μg/mL (Figure 4A,C), and the longer the treatment time, the more significant the difference. On the sixth day after infection, lesion reduction rate (LRR) of TGB treated with microminutinin reached its maximum (51.03%) (Figure 4D).

### 2.5. Effects of Microminutinin on the Mycelial Morphology of P. theae

**Figure 5:**
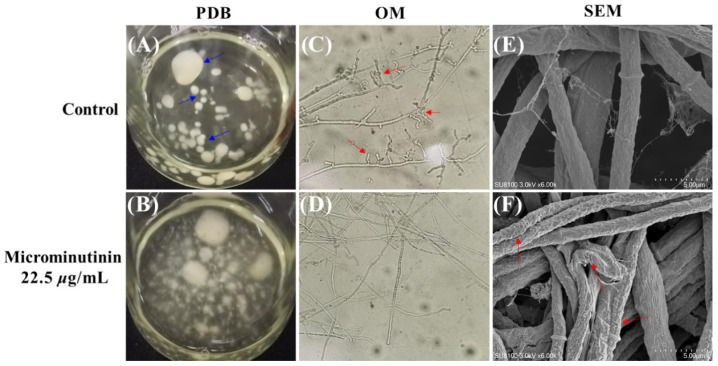
Effects of microminutinin on the mycelial morphology of *P. theae.* ((**C**,**D**): 20×). (**A**,**B**): Aggregation of *P. theae* hyphae in PDB after treatment with microcantinin; (**C**,**D**): Morphology of *P. theae* hyphae under OM after treatment with microcantinin; (**E**,**F**): Morphology of *P. theae* hyphae under SEM after treatment with microcantinin.

To observe the effect of microminutinin on hyphal morphology more clearly, this study set the concentration of 1 to 22.5 μg/mL (approximately 2 × EC_50_). From Figure 5A,B, compared with the control, after treatment with microminutinin, *P. theae* colonies were scattered in the potato dextrose broth (PDB), while the hyphae in the control clearly aggregated together to form spherical structures (blue arrow in Figure 5A).

Observing the overall and surface morphology of pathogenic hyphae through optical microscope (OM) and scanning electron microscopy (SEM) can determine whether they are growing normally. Under OM, compared with the control, the *P. theae* hyphae treated with microminutinin (22.5 μg/mL) had almost no branches (Figure 5D), while the hyphae in the control had very rich branches (red arrow in Figure 5C), which could be the main reason for the colony aggregation in the PDB control group. Further SEM observation of the mycelium surface showed that the surface of *P. theae* mycelium treated with microminutinin became rough, with cracks and severe wrinkles (red arrow in Figure 5F), while the mycelium in the control group was full and grew normally.

### 2.6. The Impact of Microminutinin on the Cell Membrane

#### 2.6.1. The Impact of Microminutinin on Cell Membrane Integrity

**Figure 6:**
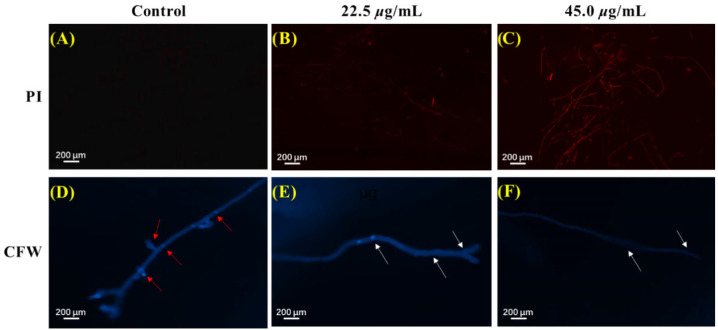
PI and CFW staining results of *P. theae* mycelium after microminutinin treatment. (**A**–**C**): PI staining results of *P. theae* hyphae after treatment with different concentrations of microminutinin; (**C**–**F**): CFW staining results of *P. theae* hyphae after treatment with different concentrations of microminutinin.

The cell plasma membrane serves as a crucial role in keeping the stability of the intracellular environment of fungi. The effect of microminutinin on the cell membrane integrity of *P. theae* mycelium can be measured using propidium iodide (PI) staining and fluorescence microscopy observation. The higher the intensity of red fluorescence, the more severe the damage caused by microminutinin to the integrity of the *P. theae* plasma membrane. As shown in Figure 6A–C, the mycelium of the control group showed almost no red fluorescence, while the mycelium exposed to microminutinin for 12 h presented strong red fluorescence with a dose effect. The above results illustrated that treatment with microminutinin could cause the loss of cell membrane integrity of *P. theae* hyphae, resulting in inhibition or even death of *P. theae* hyphae growth.

#### 2.6.2. Effects of Microminutinin on the Cell Membrane Permeability (CMP)

**Figure 7:**
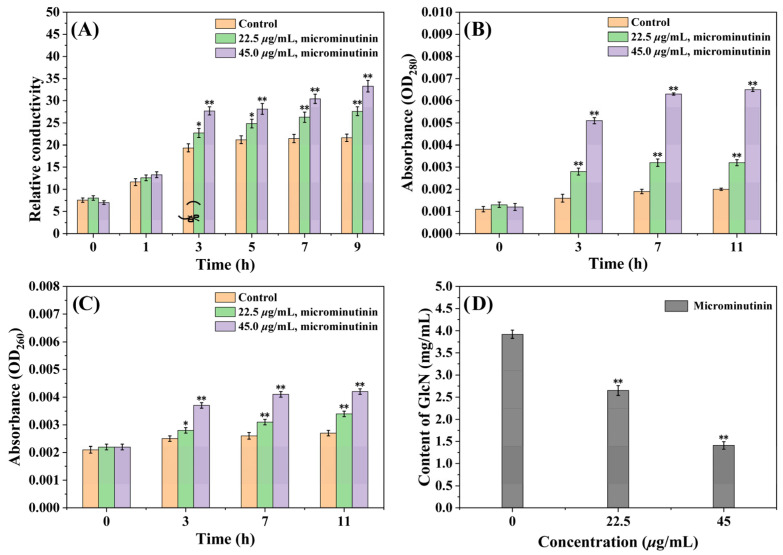
Measurement results of extracellular RC, OD_260 nm_ and OD_280 nm_ values, and cell wall chitin content of *P. theae* mycelium after microminutinin treatment. (**A**): Extracellular RC of *P. theae* after treatment with different concentrations of microminutinin; (**B**,**C**): Extracellular OD_260 nm_ and OD_280 nm_ values of *P. theae* after treatment with different concentrations of microminutinin; (**D**): GlcN content of *P. theae* after treatment with different concentrations of microminutinin. Data are the mean of three replicates, “*” on the columns represent significant difference from control (*p* < 0.05) by analysis of variance with an independent sample *t*-test, and “**” represent significant difference from control (*p* < 0.01).

CMP can reflect whether the cell membrane is damaged, and it can be evaluated by determining the extracellular conductivity value. From Figure 7A, the extracellular relative conductivity (RC) of the control group gradually increased in the first 5 h and then stabilized around 21.0%. However, the extracellular RC of the treated group with microminutinin consistently increased, with time and dose-dependent effects. After treatment with microminutinin (45.0 μg/mL) for nine hours, the RC reached its maximum of 33.28%.

Furthermore, the increase in CMP can induce the exudation of nucleic acids and proteins from cells, which can be reflected by the absorbance values (at 260 nm and 280 nm). According to Figure 7B,C, after 11 h of treatment with microminutinin, the OD_260 nm_ and OD_280 nm_ values showed a dose-and time-dependent increase, indicating the release of nucleic acids and proteins from *P. theae* cells, while the control group showed little change. After treatment with microminutinin (45.0 μg/mL) for 11 h, the OD_260 nm_ and OD_280 nm_ values reached their maximum, which were 0.0042 and 0.0065, respectively.

### 2.7. Effects of Microminutinin on the Cell Wall

The fungal cell wall, due to its highly plastic dynamic skeleton, enables cells to cope with different stresses. The impact of microminutinin on the integrity of the cell walls of *P. theae* mycelium can be detected by calcofluor white (CFW) staining and fluorescence microscopy observation. According to Figure 6D,F, all mycelia stained with CFW exhibited blue fluorescence of varying brightness. However, in the control group, the number of mycelial diaphragms was large, complete, clear, and bright, while the number and fluorescence intensity of mycelial diaphragms were significantly reduced after treatment with microminutinin, with a dose-dependent effect, indicating that microminutinin has an impact on the integrity of *P. theae* cell walls.

Chitin is a crucial substance in the formation of fungal cell walls, and changes in its content can reflect whether the fungal cell wall is destroyed. The chitin content can be reflected by the content of glucosamine (GlcN) produced by chitin degradation. In Figure 7D, the chitin content of *P. theae* mycelium treated with microminutinin showed a remarkable decrease (*p* < 0.01), with a dose-dependent effect. This result is consistent with the CFW staining results mentioned above, further proving that microminutinin has an impact on the *P. theae* cell wall.

## 3. Discussion

This study is the first to reveal the inhibitory effect of ME on plant pathogenic fungi. The results showed that at 3 mg/mL, ME had broad inhibitory effects on 12 plant pathogens (Figure 1A,B), and with the strongest inhibitory effect against *P. theae* (an IR of 68.80%), suggesting that ME has a broad-spectrum anti plant pathogenic fungal effect. Although there is currently no research on the antifungal activity of *M. euchrestifolia*, the broad-spectrum antibacterial activity of its volatile oil has been reported [17,18]. Therefore, ME may have the potential to develop botanical fungicide or bactericide.

Previous phytochemical studies have shown that *M. euchrestifolia* is rich in coumarins [17], but it is still unknown whether coumarins serve as a key role in the antifungal activity of ME. Therefore, the antifungal activity-guided combined with coumarin chromatographic feature (strong blue fluorescence under 254 nm ultraviolet lamp) guided separation method was executed in our study to isolate the key antifungal coumarins. After a series of isolation and purification steps as shown in Figure 2A, a main antifungal coumarin with broad-spectrum anti-fungal activity was isolated from the active fraction of *M. euchrestifolia* (Fr.2.3). By comparing its NMR and MS data with the data reported in the literature, compound (1) was identified as microminutinin, a fused bis-furan coumarin (Figure 4B).

The structural characteristic (Figure 4B) of microminutinin is the presence of a fused bis-furan system at positions 7 and 8. According to current research reports, microminutinin was only isolated from *Micromelum minutum*, *Micromelum falcatum*, and *Leea thoreli* plants [23,24,25,26]. However, in our report, microminutinin was obtained from the genus *Murraya* for the first time. As far as we know, there are currently no relevant reports on the biological activity of microminutinin, and this study reports for the first time its anti-plant pathogenic fungal activity. As illustrated in Figure 3A–C, microminutinin exhibited a broad repressive effect on all 8 plant pathogenic fungi tested at 100 μg/mL, with the strongest inhibitory effect on *P. theae,* with an IR of 100.00%, indicating that microminutinin had a broad-spectrum inhibitory effect on plant pathogenic fungi. Many studies have supported that some natural coumarins have excellent antibacterial activities, such as xanthotoxin [27], esculin [28], daphnetin [29], 7-Hydroxy-5,6-dimethoxycoumarin [30], and 6,8-Dihydroxy-5,7-dimethoxycoumarin [30], etc. In addition, some studies have also shown that some natural coumarins are important phytoalexins, such as scopoletin, daphnetin, esculin, and umbelliferone [31]. Interestingly, some structure-activity relationship studies [32,33] have found that the substituents at positions 7 and 8 of simple coumarins severely affect their antimicrobial activity, and microminutinin seems to conform to this structure-activity relationship. Therefore, our research results indicate that microminutinin is the main antifungal coumarin in *M. euchrestifolia* extract, with the potential to develop plant fungicides.

To further evaluate the in vivo antifungal effect of microminutinin, we tested the pathogenicity of TGB caused by *P. theae*. We first accurately calculated the EC_50_ values of microminutinin against *P. theae* using the in vitro mycelial growth rate method. From Figure 3D,E and Table 1, The EC_50_ value of microminutinin against *P. theae* was slightly higher than that of 80% carbendazim WP, indicating that microminutinin had the potential to be used as a lead compound for botanical fungicides. Subsequently, in order to ensure the smooth progress of the experiment, we selected the minimum inhibitory concentration (MIC) values of microminutinin (100 μg/mL) as the experimental concentration. The pathogenicity evaluation results showed that microminutinin significantly (*p* < 0.05) inhibited the TGB pathogenicity, and the longer the treatment time, the more significant the difference (Figure 4A,C,D), suggesting that microminutinin may have the potential for field control of TGB.

The action of fungicides can often be directly reflected in changes in the mycelium morphology [34]. In our study, we found that microminutinin treatment significantly altered the morphology of the *P. theae* mycelium, causing a severe reduction in the branching of the mycelium and leading to significant surface distortion, shrinkage, and even cracking of the mycelium. The changes in mycelial morphology suggest that microminutinin may repress the growth of *P. theae* hyphae by damaging the integrity of the membrane and/or cell wall. In recent years, there have been many reports on the destruction of mycelial structure by natural products. Yan et al. [35]. confirmed that the natural product honokiol caused the *Rhizoctonia solani* hypha to become bent, shrunk, collapsed, and deformed. In another report, 7-hydroxy-4,6-dimethyl-3H-isobenzofuran-1-one, 7-methoxy-4,6-dimethyl-3H-isobenzofuran-1-one, and 6-formyl-4-methyl-7-methoxy-3H-isobenzofuran-1-one have been found to cause different morphological alterations in the mycelium of *Alternaria alternata*, *Fusarium oxysporum*, and *Phytophthora capsica* to be different morphological alterations [36].

Fungal cell membranes serve as an important role in stabilizing the intracellular environment and other normal physiological functions [37]. We explored the antifungal mechanism of microminutinin on *P. theae* by detecting the cell membrane integrity and permeability of the treated *P. theae*. After treatment with microminutinin, the OD values of nucleic acids and proteins in the mycelial suspension, as well as the RC of the mycelial suspension, significantly increased (Figure 7A–C). Furthermore, PI staining further supported that microminutinin treatment increased the membrane permeability of *P. theae* mycelium (Figure 6A–C). Therefore, microminutinin disrupted the integrity and permeability of the *P. theae* cell membrane, causing the leakage of cellular components in the mycelium. Kayser et al. [30]. discovered that the antibacterial mechanism of coumarin compounds is closely related to the cell membrane. Lee et al. [38]. found that coumarins (umbelliferone and esculetin) can disrupt cell membrane structure and significantly impede the formation of *P. theae* biofilm.

Keeping the integrity of the cell wall composed of pectin, chitin, and galactomannan is crucial for maintaining the morphology of fungal cells and protecting them from adverse external factors [39]. In this study, we found that microminutinin treatment caused a decrease in the number and fluorescence intensity (CFW staining) of *P. theae* mycelium septa (Figure 6D,F), as well as a decrease in chitin content (Figure 7D), indicating that the integrity of *P. theae* cell walls was affected. This result is consistent with our previous study on the effect of dictamnine on *Botryosphaeria dothidea* [40]. In addition, other natural products such as citronellal on *Magnaporthe oryzae* [41] and carvacrol on *Alternaria alternata* [42] have similar effects on the cell walls of plant pathogenic fungi.

In conclusion, microminutinin has shown the potential to develop into a broad-spectrum fungicide due to its strong inhibitory effect on pathogenic fungi in plants of different families and genera. Furthermore, studies on preliminary antifungal mechanisms have shown that microminutinin exerts antifungal activity by destroying the cellular structure of plant pathogenic fungal hyphae. Importantly, this study isolated potential antifungal coumarin from *M. euchrestifolia* for the first time and elucidated its preliminary antifungal mechanisms, revealing new material sources for green prevention and control of agricultural fungal diseases. However, the main source of microminutinin currently is plants, which have low levels and are difficult to commercialize. Therefore, future research will focus on its chemical synthesis and biosynthesis to increase its yield. At the same time, further exploration of the molecular mechanism of its antifungal activity and safety assessment for crops and the environment will provide theoretical basis for its industrial application and market supervision and management.

## 4. Materials and Methods

### 4.1. Plant, Pathogens and Chemicals

Leaves and twigs of *M. euchrestifolia* were collected in September 2023 in Xishuangbanna, YunNan Province, China and air dried (humidity 8%) in a natural environment away from light to obtain airdried *M. euchrestifolia* (3.1 kg). The 12 fungal pathogens from the Key Laboratory of Fungal Resource Protection and Utilization at Jiangxi Agricultural University, including their hosts and GenBank access numbers, are *A*. *alternate*, *B*. *dothidea*, *Botryosphaeria berengeriana*, *C*. *fructicola*, *C*. *siamense*, *C*. *gloeosporioides*, *F*. *solani*, *F*. *commune*, *F*. *proliferatum*, *Fusarium oxysporum*, *Neofabraea actinidiae*, and *P*. *theae*, respectively. 80% carbendazim WP was purchased from Hubei Phosphorus City Technology Co., Ltd. (Xiaogan, China). CC and TLC were performed on 200–300 mesh silica gel and glass plates coated with GF_254_ fluorescent silica gel (Qingdao Marine Chemical, Inc., Qingdao, China), respectively. Semipreparative HPLC was performed on an instrument (Agilent 1260, XDB-C18, 5 µm, 9.4 × 250 mm, Palo Alto, CA, USA). ^1^H-(400 Hz) and ^13^C-NMR (101 Hz) were documented on a Bruker AV-400 spectrometer (Bruker, Bremerhaven, Germany). MS was recorded on an API QSTAR Pular-1 mass spectrometer (VG, Manchester, UK). HR-ESI-MS was obtained with a Bruker Daltonics, Inc. micro-TOF-Q spectrometer (VG, Manchester, UK).

### 4.2. Antifungal Coumarin Guided Isolation

Airdried *M. euchrestifolia* (3.1 kg) was extracted three times with 95% methanol (10 L each) reflux (60 °C) to obtain ME (203.2 g). At room temperature, ME was dispersed in 5 L of water and extracted sequentially with equal volumes of PE and EA. After evaporating the solvent, PF, AF, and WF were obtained. Subsequently, we evaluated the broad-spectrum antifungal effects of ME, PF, AF, and WF and selected AF with the strongest antifungal activity for antifungal coumarin guided isolation. AF (87.3 g) was added to a 200–300 mesh silica gel CC and eluted with PE/acetone (1:0, 10:1, 5:1, 3:1, 1:1, 0:1, *v*/*v*) to give six fractions (Fr.1–Fr.6). Furthermore, the antifungal activities of Fr.1–Fr.6 against *P. theae* and *B. dothidea* (most sensitive to AF) were evaluated. Based on the fluorescence and TLC display characteristics of coumarins in Fr.1–Fr.6, Fr.2 (21.8 g) was subjected to a 200–300 mesh silica gel CC with isocratic elution of PE/acetone (4:1, *v*/*v*) to give five subfractions (Fr.2.1–Fr.2.5). Furthermore, Fr.2.3 (3.1 g) was subjected to semipreparative-HPLC with isocratic elution of CH_3_OH/H_2_O (82:18, *v*/*v*; 2 mL/min; 254 nm; 23.2 min) to obtain compound 1 (54.6 mg) (Figure 2A).

*Microminutinin* (1) C_14_H_10_O_4_, Crystalline substance in MeOH, mp 114–116; ^1^H and ^13^C NMR data (400 and 100 MHz, in CDCl_3_), see SI Figures S1–S4; ESI-MS, *m/z*: 243 [M + H]^+^; HR-ESI-MS *m/z*: 243.0656 (Calculated for: [C_14_H_10_O_4_ + H]^+^, 243.0657).

### 4.3. Determination of Broad-Spectrum Antifungal Activity In Vitro

Broad-spectrum antifungal efficacy determination involved in activity-guided isolation was carried out using the solution we previously adopted [22,40,43], and 12 plant pathogens from six genera were used as the tested fungi, including *A. alternata*, *B. dothidea*, *B. berengeriana*, *C. fructicola*, *C. siamense*, *C. gloeosporioides*, *F. solani*, *F. commune*, *F. proliferatum*, *F. oxysporum*, *N. actinidiae*, *P. theae*. Briefly, each testing sample (ME, PF, AF, WF, Fr.1–Fr.6, and Fr.2.1–Fr.2.5) was uniformly dispersed in 45 °C water to generate a mother solution with a concentration of 10 mg/mL, which was then integrated into the potato dextrose agar (PDA) medium to prepare a toxic medium. The concentration of each test sample in its toxic medium is 3 mg/mL for ME, 2 mg/mL for PF, AF or WF, and 1 mg/mL for Fr.1, Fr.2, Fr.3, Fr.4, Fr.5, Fr.6, Fr.2.1, Fr.2.2, Fr.2.3, Fr.2.4 or Fr.2.5, respectively. Equal amount of distilled water was used for treatment as a control. Compound 1 was fully dissolved in acetone to prepare a stock solution, which was then integrated into 50 °C PDA medium to prepare a toxic medium containing 100 μg/mL of 1 (acetone content less than 1%). Medium containing equal volume of acetone as a control. Each bioassay process was repeated three times. Afterwards, the inoculation and cultivation of pathogenic fungi, as well as the measurement of pathogen colonization circles, were all as stated by Zhang et al. [22]. IR was calculated based on Formula (1):

(1) $$IR=\frac{Average\;diameter\;of\;the\;control-Average\;diameter\;of\;the\;treatment}{Average\;diameter\;of\;the\;control}\;\times 100\%$$

IR: Inhibition rate.

The EC_50_ values of 1 and 80% carbendazim WP (positive control drug) against *P. theae* (most sensitive to 1) were obtained based on the calculation method adopted by Song et al. [43], and the concentration gradients of 1 and 80% carbendazim WP were set to (100, 50, 25, 12.5, 6.25, 3.125) and (20, 10, 5, 2.5, 1.25, 0.5) μg/mL, respectively.

### 4.4. The Suppressive Effect of 1 on the Pathogenicity of TGB

Fresh tea leaves (“Luyun-1”) were collected from the experimental tea garden of Jiangxi Agricultural University. The acupuncture method was adopted to assess the inhibitory effect of 1 on the TGB pathogenicity. Specifically, three uniformly sized leaves without disease spots or mechanical damage were randomly selected from different tea tree shoots and used as test objects and were surface disinfected with 75% alcohol. After the tested tea leaves were punctured with a sterile syringe, a 6 mm diameter pathogen (*P. theae*) PDA disk was picked up with a sterile inoculation needle and attached with its mycelium side to the wound, and the wound was covered with a layer of degreased cotton moistened with sterile water to keep it moist. After 2 h, 50 μL of 1 solution was added to every wound at concentration of 100 μg/mL (approximately 10 × EC_50_), and an equal volume of distilled water was added to the control wound. The inoculated leaves were placed in a 26 °C incubator for cultivation, and the incidence of TGB was recorded daily. Each process was repeated 3 times. Based on the cross method to measure the diameter of lesions, LRR (%) was calculated based on Formula (2):

(2) $$LRR\left(\%\right)=\frac{Average\;diameter\;of\;the\;control-Average\;diameter\;of\;the\;treatment}{Average\;diameter\;of\;the\;control}\;\times 100\%$$

LRR: lesion reduction rate.

### 4.5. The Influence of 1 on Mycelial Morphology

#### 4.5.1. OM

OM was used to observe changes in hyphal morphology using the modified programme described by Zhao et al. [44]. Three 6 mm diameter pathogen (*P. theae*) PDA disks were added to a 25 mL PDB medium containing compound 1 with a final concentration of 22.5 μg/mL (approximately 2 × EC_50_) and cultivated at 26 °C and 160 R/min for 4 days. The PDB without compound 1 was used as a control. The mycelium was picked out and rinsed three times with distilled water. The morphological changes in the mycelium were observed under OM. Each treatment was repeated three times.

#### 4.5.2. SEM

SEM was used to observe the surface changes in the mycelium using the programme adopted by Kong et al. [45]. The 6 mm diameter pathogen (*P. theae*) PDA disks were attached to the center of PDA medium containing 1 (22.5 and 0 μg/mL) and incubated at 26 °C and 90% RH for 4 d. The next steps were as described by Kong et al. [45]. Each treatment process was repeated three times. All treated mycelial samples were examined for surface changes under SEM at (EVO-LS10, Zeiss, Germany). Each treatment was repeated three times.

### 4.6. Assay of Plasma Membrane Integrity of P. theae

Following the method adopted by Xu et al. [46], the integrity of the cytoplasmic membrane of *P. theae* mycelium was assessed by PI stain (Thermo Fisher Scientific, Waltham, MA, USA) staining with slight modifications. After being cultured in PDB medium containing 1 (0, 22.5, and 45.0 μg/mL) for 4 days, *P. theae* mycelium was harvested and dyed with 10 μg/mL PI in the dark at 37 °C for 20 min. Then, the stained mycelium was centrifuged, washed three times with 10 mmol/L phosphate-buffered saline (PBS), and observed under a fluorescence microscope (Nikon Ni-E, Tokyo, Japan). All treatments were repeated three times.

### 4.7. Cell Wall Integrity Measurement

Adopting the method we used before [40], the effect of 1 on the integrity of *P. theae* cell wall was detected through CFW stain (Sigma, St. Louis, MO, USA). The specific method is similar to that in Section 2.6 above. The harvested mycelium was stained with CFW instead of PI, and after 30 min, a 10% KOH solution was mixed into PBS. The stained samples were observed under fluorescence microscopy (Nikon Ni-U, Tokyo, Japan). Each treatment was repeated three times.

### 4.8. Determination of Electrolyte Leakage

To measure the electrolyte leakage of *P. theae*, the RC was determined following the method we previously adopted [22], with small modifications. A PDA disk with 2-day-old mycelium of *P. theae* was put into PDB medium and cultivated at 26 °C and 180 rpm for 48 h. 0.5 g of mycelium collected from PDB medium was rinsed with distilled water and moved into 20 mL of distilled water containing 1 (0, 22.5, and 45.0 μg/mL). The electric conductivity was measured at 0, 1, 3, 5, 7, and 9 h by a conductivity meter (CON510, Eutech/Oakton, Singapore). After 9 h, the conductivity of the boiled aqueous solution containing mycelium was taken as the final conductivity. All treatments were repeated three times. The RC was calculated based on Formula (3):

(3) $$Relative\;conductivity\left(\%\right)=\frac{Conductivity}{Fianal\;conductivity}\;\times 100\%$$

RC: relative conductivity

### 4.9. Cytoplasmic Leakage Assay

The leakage of cytoplasmic constituents of *P. theae* was conducted following the program adopted by Fan et al. [47]. The *P. theae* mycelium, which had been shaken in PDB medium for 48 h, was collected, washed, and transferred to 20 mL of distilled water containing 1 at 0, 22.5, and 45.0 μg/mL. After 0, 3, 7, and 11 h, the absorbance of nucleic acid (OD_260_) and protein (OD_280_) in the aqueous solution was measured, repeating each treatment three times.

### 4.10. Determination of Chitin Content

The chitin content of *P. theae* mycelium was determined by the method used by Yuan et al. [40,48] 0.5 g of dried *P. theae* mycelium cultured in PDB medium containing 1 (0, 22.5, and 45.0 μg/mL) for 48 h was hydrolyzed in 1 mL of 6 M HCl at 100 °C for 17 h. The sample without solvent was dissolved in 1 mL of water. Subsequently, 100 μL of the sample was mixed with an equal volume of 0.75 M Na_2_CO_3_ solution and incubated at 100 °C for 20 min. Finally, 0.7 mL of 95% ethanol and 100 μL of solution A (1.6 g of p-dimethylaminobenzaldehyde in 30 mL concentrated HCl and 30 mL ethanol) were added. Each treatment was repeated three times. The absorbance at 420 nm was recorded, and the chitin content was calculated based on the standard curve of glucosamine.

### 4.11. Statistical Analysis

Origin v2021 was used to generate figures and graphs. The SPSS 17.0 software was used for comprehensive statistical analysis. Significant differences among mean values (*p* < 0.05) were determined by a one-way analysis of variance with Tukey’s test. The data of the two groups were compared by an independent sample *t*-test, and the differences of *p* < 0.05 or 0.01 were statistically significant.

## 5. Conclusions

In this study, we isolated a fused bis-furan coumarin from *M. euchrestifolia* for the first time and evaluated its broad-spectrum antifungal activity against plant pathogens for the first time. The suppressive effect of microminutinin on the pathogenicity of tea gray blight caused by *P. theae* indicated its potential for field application. SEM and OM revealed that microminutinin disrupted the morphological structure of mycelium, causing hyphae to twist, shrink, and even crack and severely reducing hyphal branching. Furthermore, PI staining proved that microminutinin destroyed the integrity of the cell membrane, causing leakage of cellular components, and CFW staining and chitin content changes illustrated that microminutinin disrupted the cell wall structure. In summary, microminutinin has the potential to be developed into new plant-based fungicides or further studied as lead compounds.

## Figures and Tables

**Table 1:** Toxicities of microminutinin and carbendazim against *P. theae*.

No.	Compounds	VRE	EC_50_ (μg/mL)	R^2^	95% CI
1	microminutinin	y = 2.6466x + 2.2325	11.33	0.92	2.97–43.19
2	carbendazim	y = 1.3096x + 3.8908	7.03	0.98	4.53–10.86

95% Confidence interval (95% CI); Virulence regression equation (VRE).

## Data Availability

The original contributions presented in this study are included in the article and Supplementary Materials. Further inquiries can be directed to the corresponding authors.

## Supplementary Materials

The following supporting information can be downloaded at: https://www.mdpi.com/article/10.3390/plants14213392/s1, Figure S1: The 1H NMR spectrum of compound 1; Figure S2: The 13C-NMR spectrum of compound **1**; Figure S3: DEPT spectrum of compound **1**; Figure S4: 13C-NMR and DEPT spectrum of compound **1**; Table S1: Inhibitory effect of *M. euchrestifolia* extracts on 12 pathogenic fungi; Table S2: The inhibitory effect of MF, Fr.1 and Fr.2 on the shoot and root growth retroflexus and *D. sanguinalis*; Table S3: Inhibitory effect of subfractions from *M. euchrestifolia* extract on *B. dothidea* and *P. theae*; Table S4. Inhibitory effect of microminutinin on 8 pathogenic fungi.
