# Supplementary material for: Microminutinin, a Fused Bis-Furan Coumarin from Murraya euchrestifolia, Exhibits Strong Broad-Spectrum Antifungal Activity by Disrupting Cell Membranes and Walls
Source: Plants (Basel). 2025 Nov 5;14(21):3392. doi: 10.3390/plants14213392 (PMC12610593; doi:10.3390/plants14213392)
Supplement: Supplementary file 1 [file plants-14-03392-s001.zip › plants-3956461-supplementary (1).pdf]

Supporting Information

Content of Supplementary Materials

1. SI Figures

SI Figure S1. The ^{1}H NMR spectrum of compound **1.**

SI Figure S2. The ^{13}C-NMR spectrum of compound **1**.

SI Figure S3. DEPT spectrum of compound **1.**

SI Figure S4. ^{13}C-NMR and DEPT spectrum of compound **1**.

2. SI tables

SI table S1. Inhibitory effect of *M. euchrestifolia* extracts on 12 pathogenic fungi.

SI table S2. The inhibitory effect of MF, Fr.1 and Fr.2 on the shoot and root growth of *A. retroflexus* and *D. sanguinalis*.

SI table S3. Inhibitory effect of subfractions from *M. euchrestifolia* extract on *B. dothidea* and *P. theae*.

SI table S4. Inhibitory effect of microminutinin on 8 pathogenic fungi.

1. SI figures

SI figure S1 The ^{1}H NMR spectrum of compound **1.**

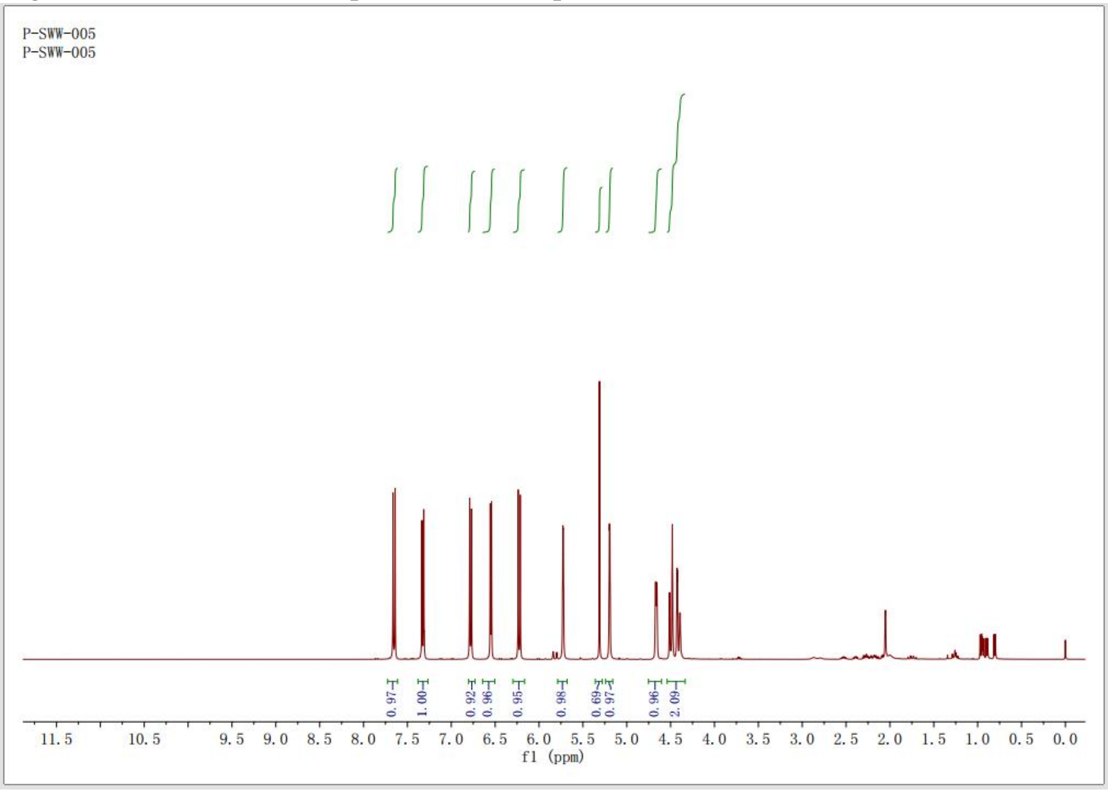

SI figure S2 The ^{13}C-NMR spectrum of compound **1**.

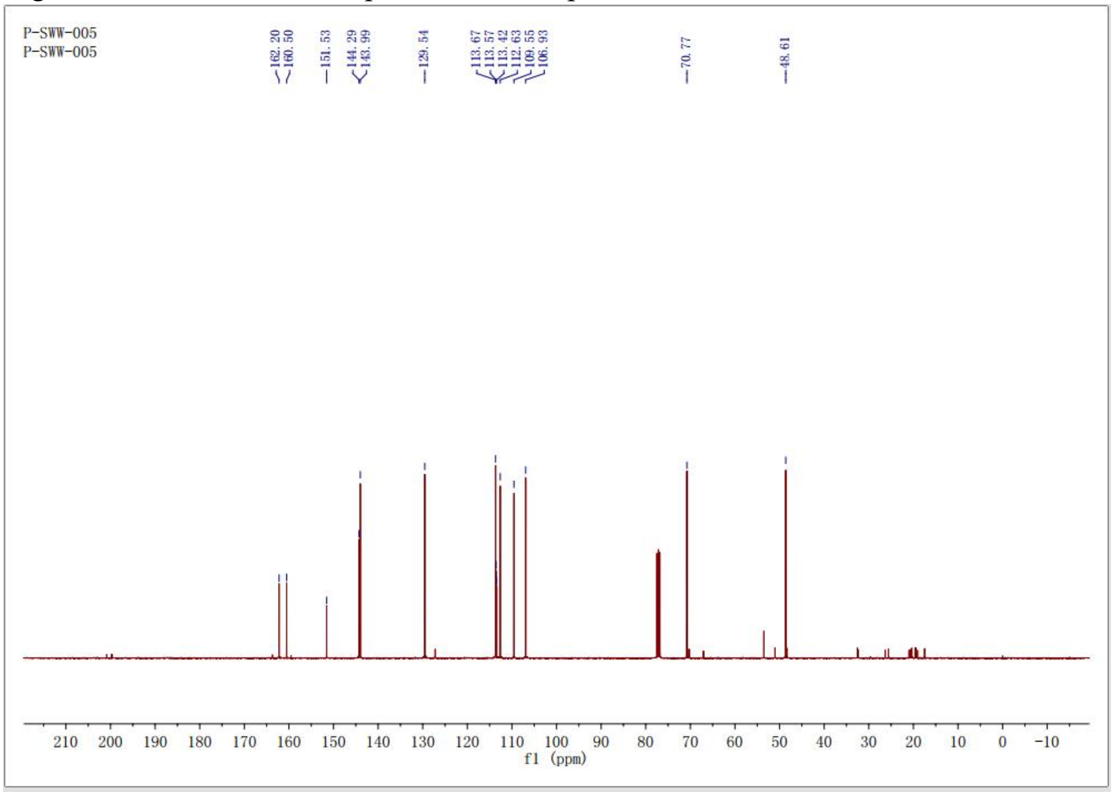

SI figure S3 DEPT spectrum of compound **1.**

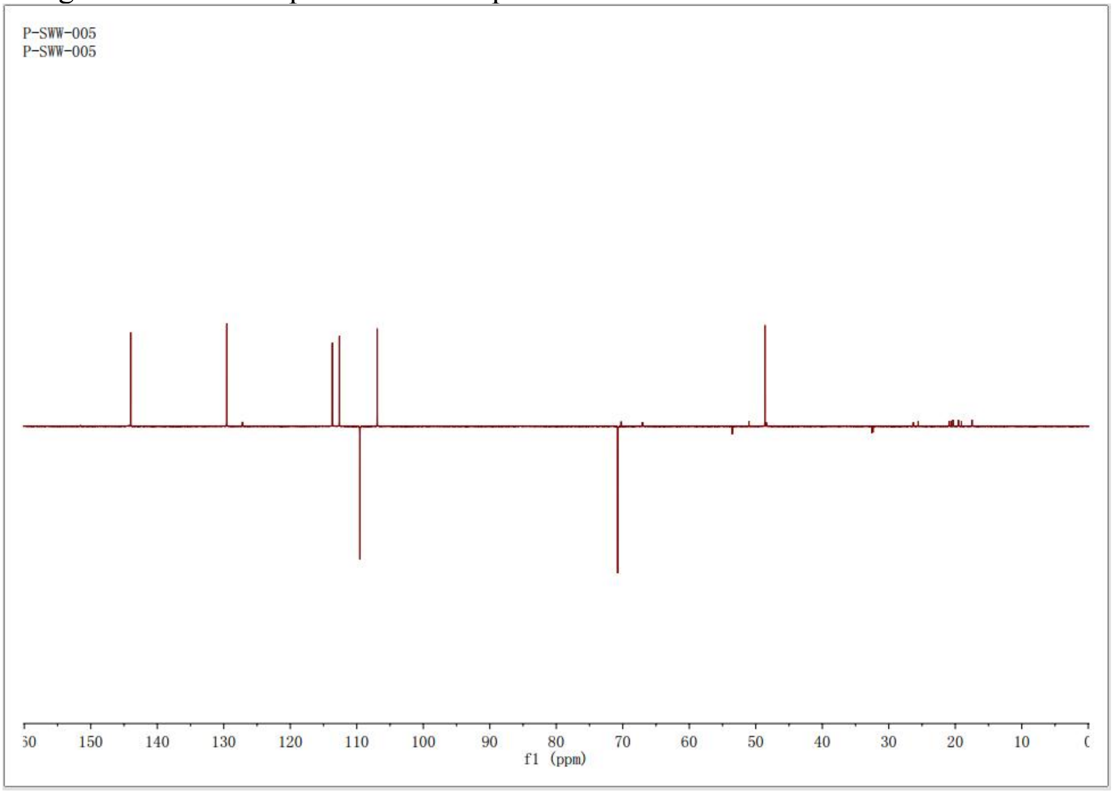

SI figure S4 ^{13}C-NMR and DEPT spectrum of compound **1**.

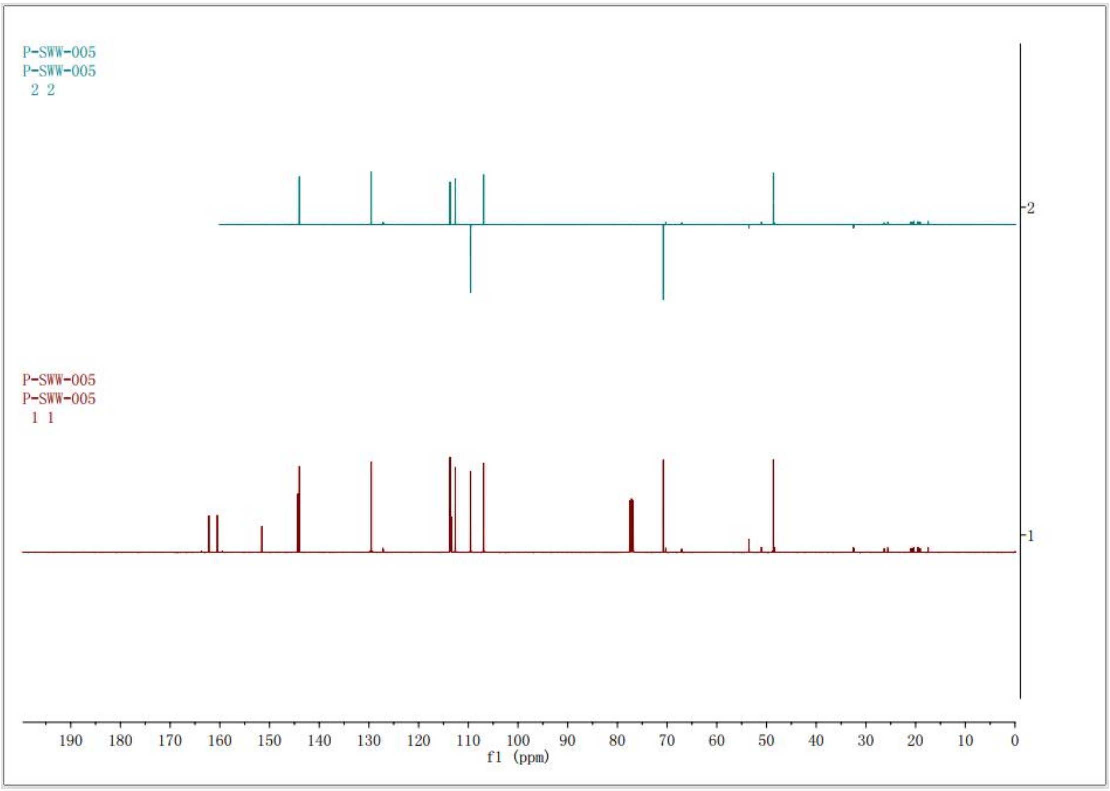

2. SI tables

SI table S1 Inhibitory effect of *M. euchrestifolia* extracts on 12 pathogenic fungi

No.	Pathogenic fungi	Inhibition rate（%）			
		ME (3 mg/mL)	PF (2 mg/mL)	AF (2 mg/mL)	WF (2 mg/mL)
1	*A. alternata*	52.70±1.91 cd	23.49±1.66 bc	59.84±2.03 cd	9.49±0.94 ef
2	*B. dothidea*	67.18±2.49 a	28.17±1.67 a	76.62±2.13 a	13.41±0.97 cd
3	*B. berengerianade*	49.60±1.63 de	19.94±1.58 ef	53.50±1.71 e	10.22±0.89 ef
4	*C. siamense*	51.29±1.72 d	21.63±1.60 de	56.75±1.81 de	14.10±1.21 bcd
5	*C. fructicola*	53.41±1.82 cd	18.17±0.90 fg	57.66±1.82 d	16.07±1.31 ab
6	*C. gloeosporioides*	46.24±1.51 e	13.40±0.99 i	49.42±1.02 f	11.91±0.89 de
7	*F. commune*	59.71±1.80 b	14.08±1.42 hi	65.68±1.83 b	13.10±1.38 cd
8	*F. oxysporum*	37.24±1.33 f	9.01±1.21 j	39.50±1.29 g	10.43±1.16 ef
9	*F. proliferatum*	33.15±1.21 g	11.32±1.14 ij	37.23±1.29 g	8.45±1.07 f
10	*F. solani*	9.14±1.05 h	7.87±1.15 j	13.37±1.07 h	8.37±1.03 f
11	*P. theae*	68.80±1.85 a	25.31±1.83 ab	78.51±2.36 a	15.43±1.54 abc
12	*S. sclerotiorum*	55.55±1.72 c	16.72±1.46 gh	61.65±1.79 c	17.15±1.47 a

ME: Methanol extract; PF: Petroleum ether extract; AF: Ethyl acetate extract; WF: aqueous phase. Data are the mean of three replicates, the different letters in SI Table 1 represent significant differences among pathogenic fungi by analysis of variance with Tukey's test at P = 0.05.

SI table S2 Inhibitory effect of fractions from *M. euchrestifolia* extract on *B. dothidea* and *P. theae*

Fractions	Inhibition rate（%）(1 mg/mL)	
	B. dothidea	*P. theae*
Fr.1	45.15±1.34 d	42.24±1.39 d
Fr.2	81.92±1.94 a	86.28±1.90 a*
Fr.3	64.82±1.48 b	67.90±1.48 b
Fr.4	51.37±1.31 c	56.22±1.46 c*
Fr.5	39.19±1.09 e	37.26±1.15 e
Fr.6	29.54±1.01 f	30.29±1.12 f
Control	0.65±0.06 g	0.60±0.05 g

Data are the mean of three replicates, the different letters on the columns represent significant differences among fractions (or subfractions) by analysis of variance with Tukey's test at P = 0.05. "*" on the columns represent significant differences between pathogenic fungi by analysis of variance with an independent sample t-test ($P < 0.05$)

SI table S3 Inhibitory effect of subfractions from *M. euchrestifolia* extracton *B. dothidea* and *P. theae*

Subfractions	Inhibition rate（%）(1 mg/mL)	
	B. dothidea	*P. theae*
Fr.2.1	22.35±0.96 e	23.23±1.01 e
Fr.2.2	71.94±2.00 b	76.29±1.94 b
Fr.2.3	85.63±2.40 a	91.13±1.92 a*
Fr.2.4	61.45±1.38 c	66.09±1.31 c*
Fr.2.5	27.40±1.12 d	31.37±1.08 d*
Control	0.60±0.05 g	0.55±0.07 g

Data are the mean of three replicates, the different letters on the columns represent significant differences among fractions (or subfractions) by analysis of variance with Tukey's test at P = 0.05. “*” on the columns represent significant differences between pathogenic fungi by analysis of variance with an independent sample t-test ($P < 0.05$).

SI table S4 Inhibitory effect of microminutinin on 8 pathogenic fungi

Pathogenic fungi	Inhibition rate（%）(100 μg/mL)
	microminutinin
A.alternata	82.99±2.63bc
B.dothidea	86.71±2.73b
C.siamense	72.12±1.82d
C.gloeosporioides	78.91±2.48c
C.fructicola	62.46±1.85e
F.commune	65.78±2.10e
F.proliferatum	51.32±1.86f
P.theae	100a

Data are the mean of three replicates, the different letters on the columns represent significant differences among pathogenic fungi by analysis of variance with Tukey's test at P = 0.05.
